# Supplementary material for: Thrombosis and thrombocytopenia after vaccination against and infection with SARS-CoV-2 in Catalonia, Spain
Source: Nat Commun. 2022 Nov 23;13:7169. doi: 10.1038/s41467-022-34669-9 (PMC9684434; doi:10.1038/s41467-022-34669-9)
Supplement: Supplementary file 1 — Supplementary Information [file 41467_2022_34669_MOESM1_ESM.pdf]

Supplementary Information

Supplementary Table 1. Characteristics of study participants stratified by age category

Characteristics of the participants in the study cohorts used for the primary analyses. Those in the general population were present in the database as of 01/01/2017. The MEDEA deprivation index is calculated at the census tract level in urban areas of Catalonia and is categorised in quintiles of deprivation. It also includes a rural category for individuals living in rural areas. *Conditions of interest: autoimmune disease, antiphospholipid syndrome, thrombophilia, asthma, atrial fibrillation, malignant neoplastic disease, diabetes mellitus, obesity, or renal impairment. †Medications of interest: non-steroidal anti-inflammatory drugs, Cox2 inhibitors, systemic corticosteroids, hormonal contraceptives, tamoxifen, and sex hormones and modulators of the genital system.

Participants aged 20 to 44

	**BNT162b2 first-dose**	**BNT162b2 second-dose**	**ChAdOx1 first-dose**	**COVID-19 diagnosis**	**General population (index date: 1st December)**
N	463,328	96,375	83,458	75,453	1,953,084
Age	40 [36 to 42]	34 [27 to 40]	35 [28 to 40]	33 [26 to 39]	35 [28 to 40]
Age: 20 to 29	41,288 (8.9%)	32,011 (33.2%)	25,029 (30.0%)	27,629 (36.6%)	572,054 (29.3%)
Age: 30 to 39	183,972 (39.7%)	35,774 (37.1%)	34,221 (41.0%)	29,023 (38.5%)	863,539 (44.2%)
Age: 40 to 49	238,068 (51.4%)	28,590 (29.7%)	24,208 (29.0%)	18,801 (24.9%)	517,491 (26.5%)
Sex: Male	216,779 (46.8%)	27,869 (28.9%)	27,760 (33.3%)	34,365 (45.5%)	997,902 (51.1%)
Years of prior observation time	15.5 [15.0 to 15.5]	15.1 [15.1 to 15.2]	15.2 [15.1 to 15.2]	14.8 [13.1 to 15.0]	11.0 [11.0 to 11.0]
Comorbidities					
Autoimmune disease	5,229 (1.1%)	1,535 (1.6%)	906 (1.1%)	752 (1.0%)	15,365 (0.8%)

Antiphospholipid syndrome	346 (0.1%)	103 (0.1%)	66 (0.1%)	33 (0.0%)	360 (0.0%)
Thrombophilia	812 (0.2%)	243 (0.3%)	152 (0.2%)	88 (0.1%)	1,170 (0.1%)
Asthma	35,833 (7.7%)	8,670 (9.0%)	7,413 (8.9%)	6,189 (8.2%)	125,023 (6.4%)
Atrial fibrillation	792 (0.2%)	140 (0.1%)	75 (0.1%)	102 (0.1%)	2,051 (0.1%)
Malignant neoplastic disease	8,364 (1.8%)	1,751 (1.8%)	1,206 (1.4%)	1,102 (1.5%)	24,040 (1.2%)
Diabetes mellitus	18,296 (3.9%)	3,429 (3.6%)	2,197 (2.6%)	2,608 (3.5%)	48,569 (2.5%)
Obesity	61,129 (13.2%)	13,786 (14.3%)	8,305 (10.0%)	11,096 (14.7%)	190,526 (9.8%)
Heart disease	17,169 (3.7%)	4,047 (4.2%)	2,602 (3.1%)	2,496 (3.3%)	47,673 (2.4%)
Hypertensive disorder	22,392 (4.8%)	3,868 (4.0%)	2,164 (2.6%)	2,903 (3.8%)	62,390 (3.2%)
Renal impairment	2,282 (0.5%)	737 (0.8%)	216 (0.3%)	378 (0.5%)	6,685 (0.3%)
COPD	1,723 (0.4%)	331 (0.3%)	180 (0.2%)	176 (0.2%)	5,285 (0.3%)
Dementia	94 (0.0%)	54 (0.1%)	5 (0.0%)	11 (0.0%)	267 (0.0%)
Medication use (183 days prior to four days prior)					
Non-steroidal anti-inflammatory drugs	104,509 (22.6%)	23,627 (24.5%)	16,150 (19.4%)	19,229 (25.5%)	471,084 (24.1%)
Cox2 inhibitors	2,043 (0.4%)	434 (0.5%)	232 (0.3%)	206 (0.3%)	4,003 (0.2%)
Systemic corticosteroids	18,139 (3.9%)	4,325 (4.5%)	2,621 (3.1%)	2,974 (3.9%)	67,536 (3.5%)

Antithrombotic and anticoagulant therapies	5,272 (1.1%)	1,116 (1.2%)	682 (0.8%)	978 (1.3%)	15,795 (0.8%)
Lipid modifying agents	2,429 (0.5%)	401 (0.4%)	152 (0.2%)	243 (0.3%)	6,253 (0.3%)
Antineoplastic and immunomodulating agents	7,211 (1.6%)	2,720 (2.8%)	1,622 (1.9%)	1,827 (2.4%)	27,194 (1.4%)
Hormonal contraceptives for systemic use	10,205 (2.2%)	3,604 (3.7%)	2,221 (2.7%)	2,672 (3.5%)	33,797 (1.7%)
Tamoxifen	70 (0.0%)	18 (0.0%)	11 (0.0%)	9 (0.0%)	251 (0.0%)
Sex hormones and modulators of the genital system	11,583 (2.5%)	3,998 (4.1%)	2,470 (3.0%)	2,946 (3.9%)	39,262 (2.0%)
One or more condition of interest	86,307 (18.6%)	19,271 (20.0%)	12,028 (14.4%)	14,392 (19.1%)	263,822 (13.5%)
One or more medication of interest	116,986 (25.2%)	27,350 (28.4%)	18,711 (22.4%)	21,753 (28.8%)	512,550 (26.2%)
One or more condition/ medication of interest	173,555 (37.5%)	39,595 (41.1%)	27,024 (32.4%)	30,693 (40.7%)	676,243 (34.6%)

Participants aged 45 to 64

	**BNT162b2 first-dose**	**BNT162b2 second-dose**	**ChAdOx1 first-dose**	**COVID-19 diagnosis**	**General population (index date: 1st December)**
N	801,005	500,199	321,024	61,478	1,550,866
Age	52 [48 to 56]	54 [51 to 57]	61 [59 to 63]	53 [48 to 58]	53 [49 to 58]
Age: 40 to 49	289,700 (36.2%)	77,075 (15.4%)	23,937 (7.5%)	19,270 (31.3%)	461,887 (29.8%)
Age: 50 to 59	482,366 (60.2%)	398,367 (79.6%)	57,038 (17.8%)	31,446 (51.2%)	776,128 (50.0%)
Age: 60 to 69	28,939 (3.6%)	24,757 (4.9%)	240,049 (74.8%)	10,762 (17.5%)	312,851 (20.2%)
Sex: Male	398,342 (49.7%)	238,465 (47.7%)	153,008 (47.7%)	29,777 (48.4%)	777,274 (50.1%)
Years of prior observation time	15.4 [15.4 to 15.4]	15.4 [15.3 to 15.5]	15.3 [15.2 to 15.3]	14.9 [14.8 to 15.1]	11.0 [11.0 to 11.0]
Comorbidities					
Autoimmune disease	14,174 (1.8%)	10,141 (2.0%)	8,151 (2.5%)	1,389 (2.3%)	28,810 (1.9%)
Antiphospholipid syndrome	518 (0.1%)	364 (0.1%)	203 (0.1%)	45 (0.1%)	415 (0.0%)

Thrombophilia	1,034 (0.1%)	669 (0.1%)	347 (0.1%)	103 (0.2%)	916 (0.1%)
Asthma	48,285 (6.0%)	29,443 (5.9%)	17,946 (5.6%)	3,926 (6.4%)	72,556 (4.7%)
Atrial fibrillation	6,446 (0.8%)	4,861 (1.0%)	6,061 (1.9%)	814 (1.3%)	15,711 (1.0%)
Malignant neoplastic disease	42,501 (5.3%)	31,472 (6.3%)	31,144 (9.7%)	4,136 (6.7%)	95,540 (6.2%)
Diabetes mellitus	59,778 (7.5%)	40,591 (8.1%)	41,502 (12.9%)	6,339 (10.3%)	140,088 (9.0%)
Obesity	165,182 (20.6%)	109,441 (21.9%)	87,616 (27.3%)	15,824 (25.7%)	308,574 (19.9%)
Heart disease	61,830 (7.7%)	44,040 (8.8%)	44,163 (13.8%)	6,011 (9.8%)	129,787 (8.4%)
Hypertensive disorder	148,009 (18.5%)	107,453 (21.5%)	107,140 (33.4%)	13,813 (22.5%)	360,110 (23.2%)
Renal impairment	12,286 (1.5%)	9,220 (1.8%)	9,815 (3.1%)	1,680 (2.7%)	29,642 (1.9%)
COPD	17,477 (2.2%)	13,277 (2.7%)	15,790 (4.9%)	1,552 (2.5%)	44,823 (2.9%)
Dementia	1,118 (0.1%)	961 (0.2%)	482 (0.2%)	148 (0.2%)	2,175 (0.1%)
Medication use (183 days prior to four days prior)					
Non-steroidal anti-inflammatory drugs	193,704 (24.2%)	121,533 (24.3%)	80,086 (24.9%)	17,333 (28.2%)	423,376 (27.3%)
Cox2 inhibitors	7,521 (0.9%)	5,249 (1.0%)	3,847 (1.2%)	542 (0.9%)	12,001 (0.8%)
Systemic corticosteroids	36,728 (4.6%)	23,721 (4.7%)	16,693 (5.2%)	3,483 (5.7%)	81,102 (5.2%)

Antithrombotic and anticoagulant therapies	15,410 (1.9%)	10,190 (2.0%)	8,929 (2.8%)	1,479 (2.4%)	31,153 (2.0%)
Lipid modifying agents	16,518 (2.1%)	12,138 (2.4%)	10,385 (3.2%)	1,171 (1.9%)	35,894 (2.3%)
Antineoplastic and immunomodulating agents	7,285 (0.9%)	4,800 (1.0%)	3,299 (1.0%)	655 (1.1%)	13,599 (0.9%)
Hormonal contraceptives for systemic use	3,809 (0.5%)	1,713 (0.3%)	343 (0.1%)	296 (0.5%)	4,973 (0.3%)
Tamoxifen	447 (0.1%)	310 (0.1%)	133 (0.0%)	36 (0.1%)	674 (0.0%)
Sex hormones and modulators of the genital system	6,125 (0.8%)	3,426 (0.7%)	1,393 (0.4%)	447 (0.7%)	8,668 (0.6%)
One or more condition of interest	241,118 (30.1%)	162,284 (32.4%)	134,534 (41.9%)	22,865 (37.2%)	480,938 (31.0%)
One or more medication of interest	208,119 (26.0%)	130,613 (26.1%)	85,991 (26.8%)	18,560 (30.2%)	450,772 (29.1%)
One or more condition/ medication of interest	367,114 (45.8%)	238,432 (47.7%)	176,719 (55.0%)	32,812 (53.4%)	747,873 (48.2%)

Participants aged 65 or older

	**BNT162b2 first-dose**	**BNT162b2 second-dose**	**ChAdOx1 first-dose**	**COVID-19 diagnosis**	**General population (index date: 1st December)**
N	757,033	730,457	187,926	37,625	1,069,544
Age	78 [73 to 84]	78 [73 to 84]	67 [66 to 68]	77 [70 to 85]	75 [69 to 82]
Age: 60 to 69	40,365 (5.3%)	34,378 (4.7%)	187,836 (100.0%)	8,261 (22.0%)	286,680 (26.8%)
Age: 70 to 79	401,584 (53.0%)	389,924 (53.4%)	86 (0.0%)	13,797 (36.7%)	428,023 (40.0%)
Age: 80 or older	315,084 (41.6%)	306,155 (41.9%)	<5	15,567 (41.4%)	354,841 (33.2%)
Sex: Male	313,787 (41.4%)	302,951 (41.5%)	89,627 (47.7%)	16,581 (44.1%)	457,916 (42.8%)
Years of prior observation time	15.3 [15.2 to 15.3]	15.3 [15.3 to 15.4]	15.3 [15.3 to 15.3]	14.9 [14.8 to 15.1]	11.0 [11.0 to 11.0]
Comorbidities					
Autoimmune disease	30,852 (4.1%)	29,669 (4.1%)	5,428 (2.9%)	1,702 (4.5%)	34,461 (3.2%)
Antiphospholipid syndrome	540 (0.1%)	525 (0.1%)	99 (0.1%)	26 (0.1%)	213 (0.0%)

Thrombophilia	1,076 (0.1%)	1,027 (0.1%)	180 (0.1%)	73 (0.2%)	622 (0.1%)
Asthma	56,166 (7.4%)	54,183 (7.4%)	10,938 (5.8%)	3,104 (8.2%)	66,856 (6.3%)
Atrial fibrillation	104,488 (13.8%)	100,524 (13.8%)	7,518 (4.0%)	6,423 (17.1%)	119,497 (11.2%)
Malignant neoplastic disease	192,806 (25.5%)	186,567 (25.5%)	27,729 (14.8%)	10,170 (27.0%)	219,053 (20.5%)
Diabetes mellitus	208,372 (27.5%)	201,300 (27.6%)	36,797 (19.6%)	11,856 (31.5%)	275,512 (25.8%)
Obesity	293,724 (38.8%)	284,056 (38.9%)	64,993 (34.6%)	15,616 (41.5%)	352,441 (33.0%)
Heart disease	329,182 (43.5%)	318,215 (43.6%)	40,652 (21.6%)	17,686 (47.0%)	388,899 (36.4%)
Hypertensive disorder	538,892 (71.2%)	521,136 (71.3%)	91,685 (48.8%)	26,838 (71.3%)	716,377 (67.0%)
Renal impairment	180,986 (23.9%)	174,741 (23.9%)	11,337 (6.0%)	10,829 (28.8%)	192,676 (18.0%)
COPD	90,561 (12.0%)	87,091 (11.9%)	14,311 (7.6%)	5,537 (14.7%)	115,592 (10.8%)
Dementia	54,058 (7.1%)	51,778 (7.1%)	711 (0.4%)	5,136 (13.7%)	69,729 (6.5%)
Medication use (183 days prior to four days prior)					
Non-steroidal anti-inflammatory drugs	217,518 (28.7%)	210,762 (28.9%)	50,996 (27.1%)	12,325 (32.8%)	365,538 (34.2%)
Cox2 inhibitors	7,800 (1.0%)	7,659 (1.0%)	2,407 (1.3%)	351 (0.9%)	10,818 (1.0%)

Systemic corticosteroids	73,841 (9.8%)	71,755 (9.8%)	12,862 (6.8%)	4,163 (11.1%)	106,964 (10.0%)
Antithrombotic and anticoagulant therapies	49,912 (6.6%)	47,079 (6.4%)	7,353 (3.9%)	3,428 (9.1%)	63,349 (5.9%)
Lipid modifying agents	27,228 (3.6%)	26,445 (3.6%)	7,802 (4.2%)	1,322 (3.5%)	38,379 (3.6%)
Antineoplastic and immunomodulating agents	11,469 (1.5%)	11,135 (1.5%)	2,090 (1.1%)	619 (1.6%)	15,733 (1.5%)
Hormonal contraceptives for systemic use	945 (0.1%)	873 (0.1%)	31 (0.0%)	119 (0.3%)	1,718 (0.2%)
Tamoxifen	196 (0.0%)	197 (0.0%)	43 (0.0%)	11 (0.0%)	282 (0.0%)
Sex hormones and modulators of the genital system	2,790 (0.4%)	2,684 (0.4%)	612 (0.3%)	199 (0.5%)	3,870 (0.4%)
One or more condition of interest	553,175 (73.1%)	534,669 (73.2%)	102,943 (54.8%)	28,692 (76.3%)	704,720 (65.9%)
One or more medication of interest	242,064 (32.0%)	234,766 (32.1%)	55,068 (29.3%)	13,639 (36.2%)	397,835 (37.2%)
One or more condition/ medication of interest	608,146 (80.3%)	588,107 (80.5%)	123,975 (66.0%)	31,290 (83.2%)	820,101 (76.7%)

Supplementary Table 2. Observed versus expected events among people vaccinated against SARS-CoV-2 or with a diagnosis of COVID-19, without requiring year of prior history

For each event of interest, the number of people contributing to the analysis from the target population, their person-years contributed, and the number of observed events are given. Expected events are estimated using indirect standardization to the general population. Standardized incidence ratios (SIRs) with 95% confidence intervals (CIs) were estimated. Events with fewer than 5 occurrences were omitted.

	N	Person-years	Observed events	Expected events	SIR (95% CI)
**Deep vein thrombosis**					
ChAdOx1 first-dose	592,808	34,054	39	43.9	0.89 (0.65 to 1.22)
BNT162b2 first-dose	2,030,008	100,883	184	177	1.04 (0.90 to 1.20)
BNT162b2 second-dose	1,329,708	78,566	130	163	0.80 (0.67 to 0.95)
**Immune thrombocytopenia**					
ChAdOx1 first-dose	593,128	34,073	12	24.9	0.48 (0.27 to 0.85)
BNT162b2 first-dose	2,031,429	100,963	97	94.5	1.03 (0.84 to 1.25)
BNT162b2 second-dose	1,330,920	78,639	61	89.1	0.68 (0.53 to 0.88)
**Ischemic stroke**					
ChAdOx1 first-dose	592,072	34,010	107	96	1.11 (0.92 to 1.35)
BNT162b2 first-dose	2,024,431	100,557	525	532.9	0.99 (0.90 to 1.07)
BNT162b2 second-dose	1,324,691	78,260	516	513.1	1.01 (0.92 to 1.10)
**Myocardial infarction**					

ChAdOx1 first-dose	592,221	34,019	72	76.5	0.94 (0.75 to 1.19)
BNT162b2 first-dose	2,028,962	100,822	281	268.1	1.05 (0.93 to 1.18)
BNT162b2 second-dose	1,328,821	78,509	272	247.9	1.10 (0.97 to 1.24)
**Myocardial infarction or ischemic stroke**					
ChAdOx1 first-dose	590,888	33,940	179	169	1.06 (0.91 to 1.23)
BNT162b2 first-dose	2,020,172	100,314	796	784	1.02 (0.95 to 1.09)
BNT162b2 second-dose	1,321,026	78,037	775	744.6	1.04 (0.97 to 1.12)
**Myocardial infarction or ischemic stroke with thrombocytopenia**					
ChAdOx1 first-dose	593,223	34,078	9	11.7	0.77 (0.40 to 1.48)
BNT162b2 first-dose	2,032,104	101,001	53	62.2	0.85 (0.65 to 1.12)
BNT162b2 second-dose	1,331,472	78,671	59	60	0.98 (0.76 to 1.27)
**Pulmonary embolism**					
ChAdOx1 first-dose	592,968	34,063	24	30.8	0.78 (0.52 to 1.16)
BNT162b2 first-dose	2,030,489	100,909	154	123.7	1.25 (1.06 to 1.46)
BNT162b2 second-dose	1,330,172	78,593	117	116.2	1.01 (0.84 to 1.21)
**Stroke**					

ChAdOx1 first-dose	591,940	34,002	111	112.3	0.99 (0.82 to 1.19)
BNT162b2 first-dose	2,023,548	100,506	583	620.7	0.94 (0.87 to 1.02)
BNT162b2 second-dose	1,323,945	78,214	588	595.9	0.99 (0.91 to 1.07)
**Stroke with thrombocytopenia**					
BNT162b2 first-dose	2,032,387	101,017	39	46.5	0.84 (0.61 to 1.15)
BNT162b2 second-dose	1,331,736	78,687	41	45	0.91 (0.67 to 1.24)
**Thrombocytopenia**					
ChAdOx1 first-dose	579,728	33,281	756	589.9	1.28 (1.19 to 1.38)
BNT162b2 first-dose	1,964,102	97,139	3,198	2,152.70	1.49 (1.43 to 1.54)
BNT162b2 second-dose	1,270,019	74,987	2,760	1,968.70	1.40 (1.35 to 1.46)
**Venous thromboembolism**					
ChAdOx1 first-dose	592,514	34,037	60	65.6	0.91 (0.71 to 1.18)
BNT162b2 first-dose	2,028,012	100,768	315	265.5	1.19 (1.06 to 1.32)
BNT162b2 second-dose	1,328,020	78,463	228	246.8	0.92 (0.81 to 1.05)
**Venous thromboembolism with thrombocytopenia**					
ChAdOx1 first-dose	593,305	34,083	8	6.2	1.28 (0.64 to 2.56)

BNT162b2 first-dose	2,032,684	101,034	27	21.7	1.24 (0.85 to 1.81)
BNT162b2 second-dose	1,332,016	78,704	20	20.4	0.98 (0.63 to 1.52)

Supplementary Table 3. Observed versus expected events among people vaccinated against SARS-CoV-2 or with a diagnosis of COVID-19, stratified by calendar time

For each event of interest, the number of people contributing to the analysis from the target population, their person-years contributed, and the number of observed events are given. Expected events are estimated using indirect standardization to the general population. Standardized incidence ratios (SIRs) with 95% confidence intervals (CIs) were estimated. Events with fewer than 5 occurrences were omitted.

	Study period	N	Person-years	Observed events	Expected events	SIR (95% CI)
**Deep vein thrombosis**						
ChAdOx1 first-dose	From March	523,617	30,078	37	41.9	0.88 (0.64 to 1.22)
BNT162b2 first-dose	Before March	255,401	14,654	36	26	1.38 (1.00 to 1.92)
BNT162b2 first-dose	From March	1,762,585	85,726	146	150.4	0.97 (0.83 to 1.14)
BNT162b2 second-dose	Before March	134,172	8,080	7	10.7	0.65 (0.31 to 1.37)
BNT162b2 second-dose	From March	1,190,254	70,172	123	151.8	0.81 (0.68 to 0.97)
**Immune thrombocytopenia**						
ChAdOx1 first-dose	From March	523,915	30,096	12	24.1	0.50 (0.28 to 0.88)
BNT162b2 first-dose	Before March	255,509	14,661	28	13.2	2.13 (1.47 to 3.08)
BNT162b2 first-dose	From March	1,763,886	85,799	69	81.1	0.85 (0.67 to 1.08)
BNT162b2 second-dose	From March	134,201	8,082	7	5.3	1.31 (0.62 to 2.75)
**Ischemic stroke**						

ChAdOx1 first-dose	Before March	67,000	3,851	5	9	0.56 (0.23 to 1.34)
ChAdOx1 first-dose	From March	522,870	30,034	101	93.2	1.08 (0.89 to 1.32)
BNT162b2 first-dose	Before March	253,789	14,560	112	84.7	1.32 (1.10 to 1.59)
BNT162b2 first-dose	From March	1,758,645	85,495	409	445.8	0.92 (0.83 to 1.01)
BNT162b2 second-dose	Before March	133,432	8,036	31	31.5	0.98 (0.69 to 1.40)
BNT162b2 second-dose	From March	1,185,997	69,912	484	479.5	1.01 (0.92 to 1.10)
**Myocardial infarction**						
ChAdOx1 first-dose	From March	523,018	30,043	71	73.3	0.97 (0.77 to 1.22)
BNT162b2 first-dose	Before March	255,458	14,658	41	38.8	1.06 (0.78 to 1.44)
BNT162b2 first-dose	From March	1,761,492	85,663	239	228.5	1.05 (0.92 to 1.19)
BNT162b2 second-dose	Before March	134,279	8,086	18	16.2	1.11 (0.70 to 1.77)
BNT162b2 second-dose	From March	1,189,264	70,111	254	231.1	1.10 (0.97 to 1.24)
**Myocardial infarction or ischemic stroke**						
ChAdOx1 first-dose	Before March	66,990	3,850	6	14	0.43 (0.19 to 0.95)
ChAdOx1 first-dose	From March	521,699	29,965	172	163.1	1.05 (0.91 to 1.22)
BNT162b2 first-dose	Before March	253,263	14,529	150	120.7	1.24 (1.06 to 1.46)
BNT162b2 first-dose	From March	1,754,933	85,285	643	660	0.97 (0.90 to 1.05)

BNT162b2 second-dose	Before March	133,302	8,028	46	46.6	0.99 (0.74 to 1.32)
BNT162b2 second-dose	From March	1,182,475	69,698	728	695.3	1.05 (0.97 to 1.13)
**Myocardial infarction or ischemic stroke with thrombocytopenia**						
ChAdOx1 first-dose	From March	524,007	30,101	8	11.4	0.70 (0.35 to 1.40)
BNT162b2 first-dose	Before March	255,692	14,672	17	9.7	1.75 (1.09 to 2.82)
BNT162b2 first-dose	From March	1,764,382	85,826	36	52.3	0.69 (0.50 to 0.95)
BNT162b2 second-dose	From March	1,191,875	70,269	56	56.2	1.00 (0.77 to 1.29)
**Pulmonary embolism**						
ChAdOx1 first-dose	From March	523,765	30,087	22	29.7	0.74 (0.49 to 1.12)
BNT162b2 first-dose	Before March	255,440	14,657	31	18	1.72 (1.21 to 2.45)
BNT162b2 first-dose	From March	1,763,026	85,750	123	105.2	1.17 (0.98 to 1.40)
BNT162b2 second-dose	Before March	134,183	8,081	9	7.2	1.25 (0.65 to 2.40)
BNT162b2 second-dose	From March	1,190,707	70,199	107	108.5	0.99 (0.82 to 1.19)
**Stroke**						
ChAdOx1 first-dose	Before March	66,996	3,850	5	10.7	0.47 (0.19 to 1.12)
ChAdOx1 first-dose	From March	522,745	30,026	105	108.8	0.97 (0.80 to 1.17)

BNT162b2 first-dose	Before March	253,562	14,547	127	98.4	1.29 (1.08 to 1.54)
BNT162b2 first-dose	From March	1,757,992	85,458	450	519.5	0.87 (0.79 to 0.95)
BNT162b2 second-dose	Before March	133,361	8,032	36	36.8	0.98 (0.71 to 1.36)
BNT162b2 second-dose	From March	1,185,324	69,871	551	556.7	0.99 (0.91 to 1.08)
**Stroke with thrombocytopenia**						
BNT162b2 first-dose	Before March	255,749	14,675	13	7.5	1.74 (1.01 to 2.99)
BNT162b2 first-dose	From March	1,764,605	85,839	26	38.9	0.67 (0.46 to 0.98)
BNT162b2 second-dose	From March	1,192,127	70,285	39	42	0.93 (0.68 to 1.27)
**Thrombocytopenia**						
ChAdOx1 first-dose	Before March	66,560	3,825	29	50.2	0.58 (0.40 to 0.83)
ChAdOx1 first-dose	From March	510,974	29,331	725	559.4	1.30 (1.21 to 1.39)
BNT162b2 first-dose	Before March	242,978	13,927	612	315.7	1.94 (1.79 to 2.10)
BNT162b2 first-dose	From March	1,709,196	82,714	2,574	1,829.70	1.41 (1.35 to 1.46)
BNT162b2 second-dose	Before March	129,268	7,783	244	134.8	1.81 (1.60 to 2.05)
BNT162b2 second-dose	From March	1,135,530	66,895	2,505	1,828.50	1.37 (1.32 to 1.42)
**Venous thromboembolism**						
ChAdOx1 first-dose	From March	523,332	30,061	56	62.9	0.89 (0.69 to 1.16)

BNT162b2 first-dose	Before March	254,999	14,631	63	39.1	1.61 (1.26 to 2.06)
BNT162b2 first-dose	From March	1,761,000	85,636	250	225.5	1.11 (0.98 to 1.25)
BNT162b2 second-dose	Before March	134,008	8,070	14	15.9	0.88 (0.52 to 1.48)
BNT162b2 second-dose	From March	1,188,739	70,081	213	230.2	0.93 (0.81 to 1.06)
**Venous thromboembolism with thrombocytopenia**						
ChAdOx1 first-dose	From March	524,089	30,106	7	6.1	1.15 (0.55 to 2.41)
BNT162b2 first-dose	From March	1,764,790	85,849	23	18.6	1.24 (0.82 to 1.86)
BNT162b2 second-dose	From March	1,192,373	70,300	19	19.1	0.99 (0.63 to 1.56)

Supplementary Table 4. Observed versus expected events among people vaccinated against SARS-CoV-2 or with a diagnosis of COVID-19, excluding vaccinated persons with a history of COVID-19

For each event of interest, the number of people contributing to the analysis from the target population, their person-years contributed, and the number of observed events are given. Expected events are estimated using indirect standardization to the general population. Standardized incidence ratios (SIRs) with 95% confidence intervals (CIs) were estimated. Events with fewer than 5 occurrences were omitted.

	N	Person-years	Observed events	Expected events	SIR (95% CI)
**Deep vein thrombosis**					
ChAdOx1 first-dose	592,808	34,054	39	43.9	0.89 (0.65 to 1.22)
BNT162b2 first-dose	2,030,008	100,883	184	177	1.04 (0.90 to 1.20)
BNT162b2 second-dose	1,329,708	78,566	130	163	0.80 (0.67 to 0.95)
**Immune thrombocytopenia**					
ChAdOx1 first-dose	593,128	34,073	12	24.9	0.48 (0.27 to 0.85)
BNT162b2 first-dose	2,031,429	100,963	97	94.5	1.03 (0.84 to 1.25)
BNT162b2 second-dose	1,330,920	78,639	61	89.1	0.68 (0.53 to 0.88)
**Ischemic stroke**					
ChAdOx1 first-dose	592,072	34,010	107	96	1.11 (0.92 to 1.35)
BNT162b2 first-dose	2,024,431	100,557	525	532.9	0.99 (0.90 to 1.07)
BNT162b2 second-dose	1,324,691	78,260	516	513.1	1.01 (0.92 to 1.10)

**Myocardial infarction**					
ChAdOx1 first-dose	592,221	34,019	72	76.5	0.94 (0.75 to 1.19)
BNT162b2 first-dose	2,028,962	100,822	281	268.1	1.05 (0.93 to 1.18)
BNT162b2 second-dose	1,328,821	78,509	272	247.9	1.10 (0.97 to 1.24)
**Myocardial infarction or ischemic stroke**					
ChAdOx1 first-dose	590,888	33,940	179	169	1.06 (0.91 to 1.23)
BNT162b2 first-dose	2,020,172	100,314	796	784	1.02 (0.95 to 1.09)
BNT162b2 second-dose	1,321,026	78,037	775	744.6	1.04 (0.97 to 1.12)
**Myocardial infarction or ischemic stroke with thrombocytopenia**					
ChAdOx1 first-dose	593,223	34,078	9	11.7	0.77 (0.40 to 1.48)
BNT162b2 first-dose	2,032,104	101,001	53	62.2	0.85 (0.65 to 1.12)
BNT162b2 second-dose	1,331,472	78,671	59	60	0.98 (0.76 to 1.27)
**Pulmonary embolism**					
ChAdOx1 first-dose	ChAdOx1 first-dose	592,968	34,063	24	30.8
BNT162b2 first-dose	BNT162b2 first-dose	2,030,489	100,909	154	123.7
BNT162b2 second-dose	BNT162b2 second-dose	1,330,172	78,593	117	116.2
**Stroke**					

ChAdOx1 first-dose	591,940	34,002	111	112.3	0.99 (0.82 to 1.19)
BNT162b2 first-dose	2,023,548	100,506	583	620.7	0.94 (0.87 to 1.02)
BNT162b2 second-dose	1,323,945	78,214	588	595.9	0.99 (0.91 to 1.07)
**Stroke with thrombocytopenia**					
BNT162b2 first-dose	2,032,387	101,017	39	46.5	0.84 (0.61 to 1.15)
BNT162b2 second-dose	1,331,736	78,687	41	45	0.91 (0.67 to 1.24)
**Thrombocytopenia**					
ChAdOx1 first-dose	579,728	33,281	756	589.9	1.28 (1.19 to 1.38)
BNT162b2 first-dose	1,964,102	97,139	3,198	2,152.70	1.49 (1.43 to 1.54)
BNT162b2 second-dose	1,270,019	74,987	2,760	1,968.70	1.40 (1.35 to 1.46)
**Venous thromboembolism**					
ChAdOx1 first-dose	592,514	34,037	60	65.6	0.91 (0.71 to 1.18)
BNT162b2 first-dose	2,028,012	100,768	315	265.5	1.19 (1.06 to 1.32)
BNT162b2 second-dose	1,328,020	78,463	228	246.8	0.92 (0.81 to 1.05)
**Venous thromboembolism with thrombocytopenia**					
ChAdOx1 first-dose	593,305	34,083	8	6.2	1.28 (0.64 to 2.56)
BNT162b2 first-dose	2,032,684	101,034	27	21.7	1.24 (0.85 to 1.81)

BNT162b2 second-dose	1,332,016	78,704	20	20.4	0.98 (0.63 to 1.52)
